# Understanding Pulmonary Autograft Remodeling After the Ross Procedure: Stick to the Facts

**DOI:** 10.3389/fcvm.2022.829120

**Published:** 2022-02-09

**Authors:** Lucas Van Hoof, Peter Verbrugghe, Elizabeth A. V. Jones, Jay D. Humphrey, Stefan Janssens, Nele Famaey, Filip Rega

**Affiliations:** ^1^Department of Cardiac Surgery, University Hospitals Leuven, Leuven, Belgium; ^2^Center for Molecular and Vascular Biology, KU Leuven, Leuven, Belgium; ^3^Department of Biomedical Engineering, Yale University, New Haven, CT, United States; ^4^Department of Cardiology, University Hospitals Leuven, Leuven, Belgium; ^5^Biomechanics Section, KU Leuven, Leuven, Belgium

**Keywords:** Ross procedure, pulmonary autograft, mechanobiology, remodeling, extracellular matrix, external support

## Abstract

The Ross, or pulmonary autograft, procedure presents a fascinating mechanobiological scenario. Due to the common embryological origin of the aortic and pulmonary root, the conotruncus, several authors have hypothesized that a pulmonary autograft has the innate potential to remodel into an aortic phenotype once exposed to systemic conditions. Most of our understanding of pulmonary autograft mechanobiology stems from the remodeling observed in the arterial wall, rather than the valve, simply because there have been many opportunities to study the walls of dilated autografts explanted at reoperation. While previous histological studies provided important clues on autograft adaptation, a comprehensive understanding of its determinants and underlying mechanisms is needed so that the Ross procedure can become a widely accepted aortic valve substitute in select patients. It is clear that protecting the autograft during the early adaptation phase is crucial to avoid initiating a sequence of pathological remodeling. External support in the freestanding Ross procedure should aim to prevent dilatation while simultaneously promoting remodeling, rather than preventing dilatation at the cost of vascular atrophy. To define the optimal mechanical properties and geometry for external support, the ideal conditions for autograft remodeling and the timeline of mechanical adaptation must be determined. We aimed to rigorously review pulmonary autograft remodeling after the Ross procedure. Starting from the developmental, microstructural and biomechanical differences between the pulmonary artery and aorta, we review autograft mechanobiology in relation to distinct clinical failure mechanisms while aiming to identify unmet clinical needs, gaps in current knowledge and areas for further research. By correlating clinical and experimental observations of autograft remodeling with established principles in cardiovascular mechanobiology, we aim to present an up-to-date overview of all factors involved in extracellular matrix remodeling, their interactions and potential underlying molecular mechanisms.

## Introduction

The aortic valve opens and closes continuously, upwards of 100,000 times per day. Smooth functioning of the valve throughout a lifetime is enabled by its innate remodeling ability (1). Yet, the valve may need to be replaced in cases of unrepairable aortic valve disease, as, for example, in the setting of a bicuspid aortic valve. Especially in young adults with a long life expectancy, an aortic valve substitute should optimally restore aortic root biomechanics and hemodynamic function.

**Figure 1:**
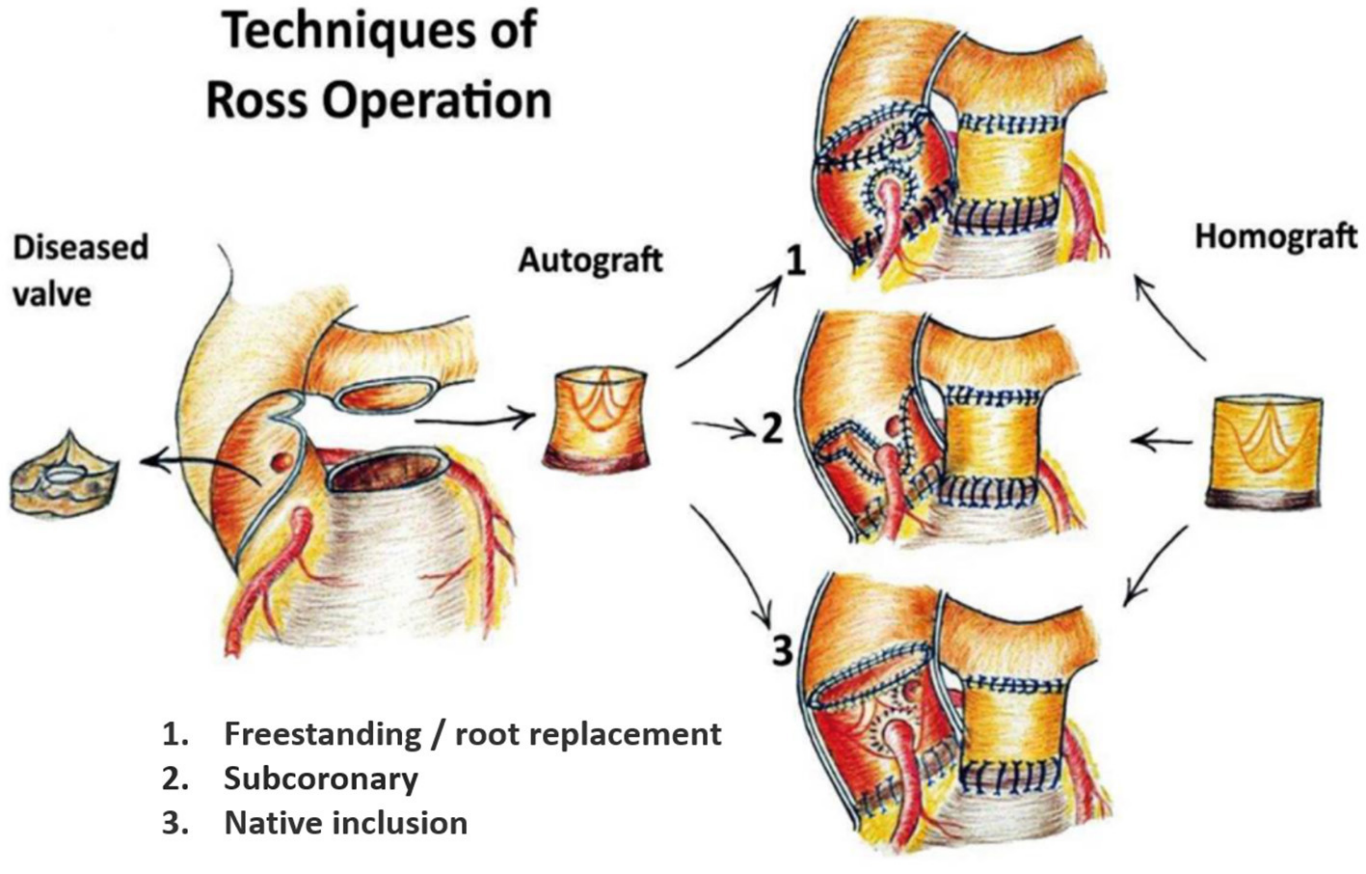
The 3 main techniques of the Ross procedure. 1. Freestanding root replacement technique with implantation of the entire pulmonary root into the left ventricular outflow tract. 2. Subcoronary technique: implantation of the pulmonary valve only within the native aortic annulus. 3. Autologous/native inclusion technique with implantation of the pulmonary autograft within the native aortic wall to prevent dilatation. Figure reproduced from Sievers (4), journal ceased publication no permission could be requested.

Disappointing outcomes of the first prosthetic valves led to the search for biological alternatives and in 1962, Donald N. Ross commenced implanting aortic valve homografts in patients (2). The lack of availability of homografts in all sizes, the lack of growth potential in children, and their limited durability prompted the quest for a living valve alternative (3). In the Ross procedure, first performed in a patient in 1967, the diseased aortic valve is replaced by the patient's own pulmonary valve and a pulmonary homograft is implanted in the pulmonary position (Figure 1) (5). As the so-called pulmonary autograft is a native tissue substitute, it offers an excellent hemodynamic profile and resistance to endocarditis without the need for anticoagulant therapy (6, 7). This translates into superior exercise capacity and freedom from valve-related complications when compared to mechanical or bioprosthetic valve replacement (8–10). Therefore, the Ross procedure is the only aortic valve replacement that can restore long-term survival and quality-of-life to that of the age-matched population (11–13). Yet, due to a perceived risk of increased operative mortality and the fear of complex reoperations on two valves, there remains skepticism toward the procedure (14, 15).

The Ross procedure presents a fascinating mechanobiological scenario. After devascularizing the autograft, it is placed in the systemic circulation and suddenly exposed to a five- to eight-fold greater blood pressure. In the freestanding root technique (Figure 1.1), the pulmonary autograft often dilates immediately (16). Nevertheless, many patients have a well-functioning neo-aortic valve multiple decades post-operatively, indicating a living, remodeling valve (17, 18). As the aorta and pulmonary root share a common embryological origin, the conotruncus, several authors have hypothesized that the pulmonary autograft has the innate ability to remodel into an aortic phenotype (19–22). Unfortunately, there are few data on successfully remodeled tissue. Rather, most *ex vivo* studies on human autografts evaluated tissue acquired at reoperation and even detailed histological reports of well-functioning autografts are scarce. Therefore, the adaptive mechanisms of the pulmonary autograft are poorly understood. It appears, however, that stress-shielding the pulmonary autograft using meticulous surgical technique, blood pressure control, or external support may further promote adaptation to the systemic circulation (19, 23).

Previous clinical studies and animal models have shown an increase in collagen in both autograft walls and leaflets (24–26). Remodeling of collagen, a key mechanism in cardiovascular biology that can increase tensile stiffness and strength, is also seen in pulmonary hypertension, systemic hypertension and aortic aneurysms (27–29). Therefore, it is likely an essential component of successful remodeling after the Ross procedure. Additional mechanisms such as an increase in cell-extracellular matrix (ECM) connections or collagen cross-linking may contribute to autograft remodeling, but have not yet been identified for the Ross procedure. Proteomic characterization of dilated autografts suggests a unique (mal)adaptive process that differs from that of ascending aortic aneurysms (30). Because all mechanisms aiming to restore tissue stress levels have the greatest chance of succeeding before overt dilatation, the early remodeling phase appears crucial. Furthermore, it is uncertain whether the pulmonary valve has a greater inherent remodeling ability than the wall, or if the wall is just more likely to suffer maladaptation in the freestanding Ross procedure because it sits unrestrained.

Prior reviews on the Ross procedure have focused mainly on patient selection and surgical technique yet speculate about autograft remodeling (19, 23). Nevertheless, long-term success of the Ross procedures relies in the first place on a living, remodeling autograft. Therefore, a comprehensive understanding of autograft adaptation and its determinants is needed so that we can identify patient-specific strategies to promote remodeling and make the pulmonary autograft a permanent aortic valve substitute. Starting with a comparison between the pulmonary artery and aorta, we review autograft mechanobiology in relation to the distinct clinical failure mechanisms. Furthermore, we evaluate the evidence regarding strategies to promote pulmonary autograft adaptation. By correlating clinical and experimental observations of autograft remodeling with established principles in cardiovascular mechanobiology, we aim to present an overview of factors involved in ECM remodeling and their interactions. Simultaneously, we aim to indicate unmet clinical needs, gaps in current knowledge, and areas for further research.

## Surgical Techniques of the Ross Procedure

The Ross procedure was first performed with the scalloped pulmonary autograft implanted in subcoronary position, avoiding the need for coronary reimplantation (Figure 1.2) (31). The freestanding root replacement technique was introduced in 1974, once reimplantation of the coronary arteries became technically feasible (3). As this iteration is more reproducible and applicable over a wide range of anatomies, it is the most commonly used today (32). Furthermore, this technique enables neo-aortic root expansion during somatic growth in children.

**Figure 2:**
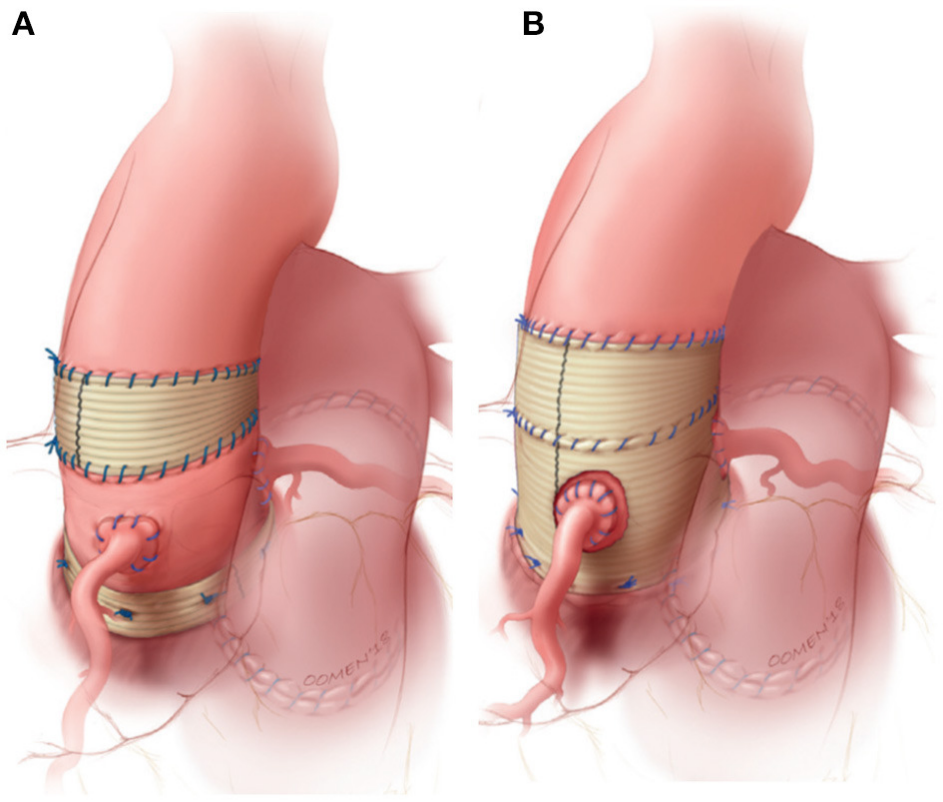
Most commonly used strategies to externally support the freestanding pulmonary autograft. **(A)** External subvalvular annuloplasty and sinotubular junction (STJ) stabilization in patients with risk factors for autograft dilatation. **(B)** Wrapping of the entire autograft within a cylinder of vascular tube graft. Figure adapted with permission from Mazine et al. (19).

Upon realizing the risk of dilatation with the root technique, the autologous inclusion technique was introduced whereby the autograft is included within the native aortic wall (Figure 1.3). Although this technique reliably prevents dilatation, it is not applicable in cases of severe size mismatch (16, 18, 33). Prosthetic external support also has the potential to prevent neo-aortic dilatation and is nowadays most commonly performed by placing the autograft within a cylinder of Dacron vascular graft (Figure 2B) (34–36). The long-term effect on reoperation rate and mechanobiological adaptation have yet to be determined (13). The use of external subvalvular annuloplasty and sinotubular junction (STJ) stabilization in patients with an enlarged annulus or STJ was introduced in a systematic fashion by Ismail El-Hamamsy (Figure 2A) (19). Using this “tailored approach” combined with strict blood pressure control, it appears possible to mitigate dilatation while avoiding the potentially deleterious hemodynamic and histological effects of total external support (37).

## Clinical Failure Mechanisms

**Figure 3:**
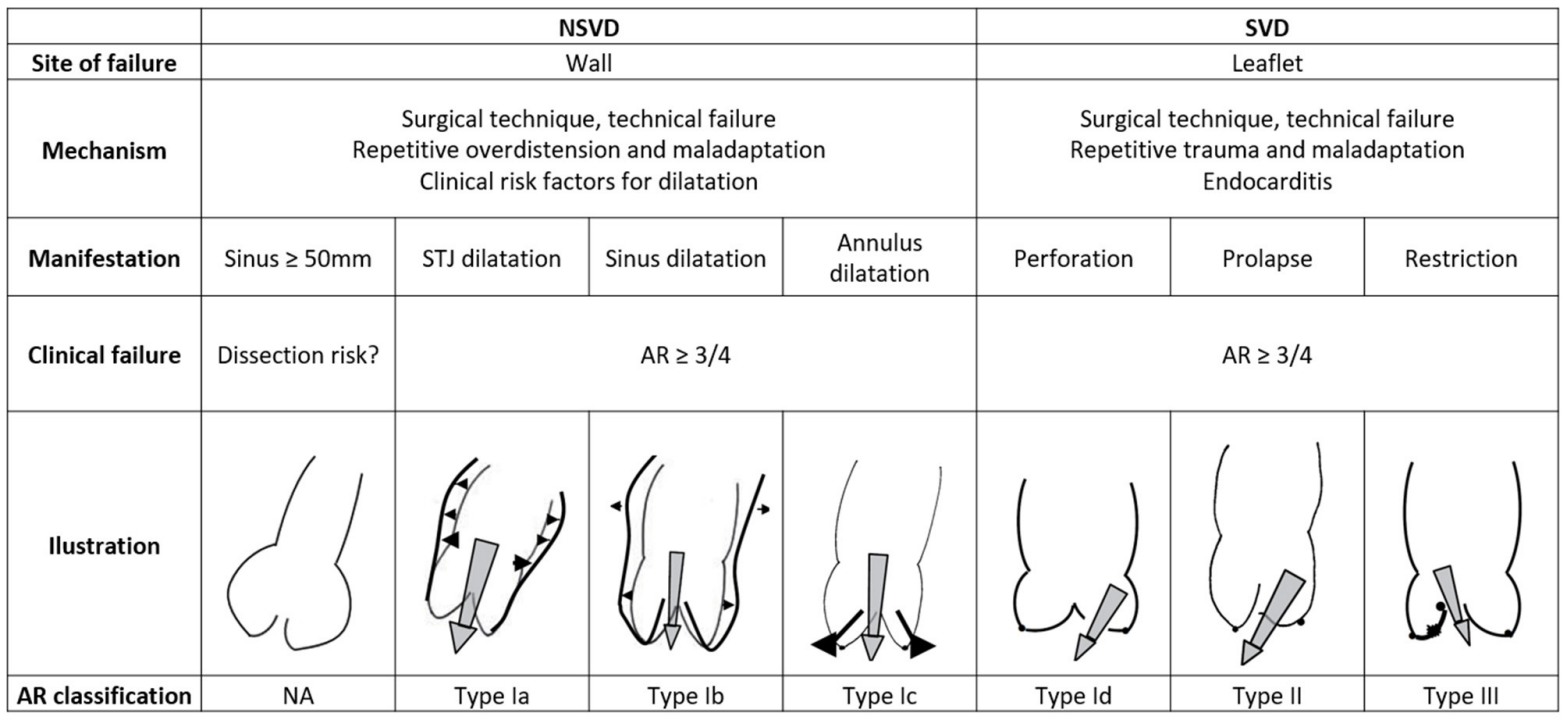
Classification of the failure mechanisms of the Ross procedure and correlation with El Khoury's functional classification of aortic regurgitation. AR, aortic regurgitation; SVD, structural valvular degeneration; NSVD, non-structural valvular degeneration. Illustrations adapted with permission from Boodhwani et al. (39).

Clinical autograft failure, and subsequent reoperation, can be related to wall dilatation, leaflet degeneration, or both. Occurring exclusively after the root replacement technique, non-structural valve degeneration is defined as greater than moderate aortic regurgitation (AR ≥ 3/4) caused by dilatation or autograft wall dilatation beyond 50 mm, with or without associated AR (38). Structural valve degeneration, defined as greater than moderate AR caused by leaflet degeneration or prolapse, is the most common mode of failure for the subcoronary technique, yet it can occur in all variations of the Ross procedure (38). An overview of possible failure patterns, based on anatomical site and underlying mechanism, and correlation with the functional classification of AR as proposed by the group of El Khoury is shown in Figure 3 (39). As the wall and leaflets can degenerate concomitantly, combinations of the described mechanisms are possible, depending on the specific failure phenotype.

In expert hands, durability of the subcoronary technique may exceed that of the root replacement technique, without the risk of dilatation. As a leader in his field, Dr. Hans-Hinrich Sievers reports excellent freedom from reoperation of 89.8% at 20 years post-operatively (17). On the other hand, by maximally respecting leaflet anatomy and relations, the root replacement technique has early superiority over the subcoronary and inclusion techniques (18). For any technique, imperfect surgical implantation may result in early AR, progressive in nature due to increased leaflet stress, and potentially lead to early technical failure.

For the root replacement technique, initial elastic dilatation occurs immediately upon release of the aortic cross-clamp due to the pulmonary artery's compliance (**Figure 5**) (40, 41). Furthermore, up to 60% of the dilatation that is present at 1 year manifested prior to hospital discharge (42). An intuitive hypothesis dictates that in patients with pronounced early dilatation, in itself leading to thinning of the wall, a cycle of pathological remodeling is initiated as the autograft is not permitted to adapt; dilatation begets dilatation (37). Progressive annular or STJ dilatation is known to cause leaflet malcoaptation with a central regurgitant jet (Figure 3) (39, 43, 44). Isolated autograft sinus dilatation on the other hand, is less likely to lead to AR (45).

Although chronic dilatation, related to tissue remodeling, appears to be a slow process in most patients, autograft diameter will exceed 40 mm in up to 50% of patients at 12 years post-operatively (33, 46). At 15 years, up to 24% of patients will require a reoperation for non-structural valve degeneration (13, 32, 38, 47). For the root replacement technique, risk factors for dilatation and subsequent reoperation are pre-operative isolated AR, a large aortic annulus, size mismatch between aortic and pulmonary annulus, pre-existing aortic dilatation, younger age, male sex and post-operative hypertension (13, 32, 38, 47, 48). These variables should be kept in mind when selecting candidates for the Ross procedure (19).

While less straightforward than for genetically determined aortic aneurysms, there appears to be an association between autograft diameter and dissection. Pulmonary autograft dissection has been described in at least 9 cases, occurring between 5 and 18 years after index surgery at an autograft diameter of 54–64 mm (49–57). All patients presented with aortic insufficiency related to a bicuspid aortic valve (BAV) at initial operation. Furthermore, a common feature was pronounced early or sudden dilatation, for example during pregnancy, indicating compromised mechanical homeostasis. In 6 out of 9 cases, the dissection originated in the non-coronary cusp, possibly related to elevated local wall stress. As all dissections were localized without crossing suture-lines, most were incidental findings on imaging, and histopathological assessment confirmed the subacute-to-chronic nature of these dissections (51, 53, 54). In one case, the non-coronary sinus ruptured (56). The critical size threshold for reoperation, when the risk of dissection exceeds the risk of reoperation, is still uncertain. It seems that the risk of autograft dissection or rupture is very low for a diameter below 50 mm, indicating that the decision to reoperate for dilatation should be tailored individually.

## Autograft Characteristics

A thorough understanding of pulmonary autograft mechanobiology begins with studying the differences between the pulmonary and aortic root. As both vessels have the same embryological origin, the conotruncus, their basic histological composition and anatomy are initially similar (58). Due to diverging hemodynamic conditions post-natally, after the ductus arteriosus closes, the aorta and pulmonary artery and their respective valves develop distinct microstructural and mechanical properties (59).

### Surgical Anatomy

**Figure 4:**
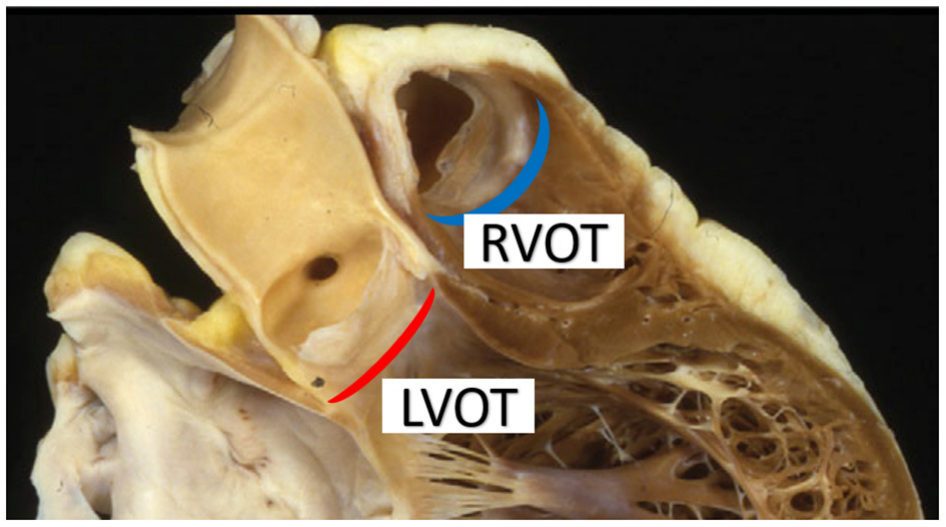
The aortic annulus (red crescent) is embedded within the fibrous skeleton of the heart whereas the pulmonary annulus (blue crescent) consists of a freestanding rim of infundibular muscle lifting the pulmonary leaflets away from the interventricular septum. LVOT, left ventricular outflow tract; RVOT, right ventricular outflow tract. Figure adapted with permission from Ho (60).

Both arteries are said to arise from an “annulus,” but the shape of the ventriculo-aortic junction does not constitute a true circle. The aortic root has a crown like band of fibrous tissue at its base with the ventriculo-aortic junction embedded centrally in the heart and inserted on both the atrioventricular valves and the thick left ventricular myocardium (Figure 4). The pulmonary root on the other hand, has no fibrous annulus and originates from the right ventricular infundibulum, a freestanding rim of muscle seated on the right ventricle and septum (61). Both valves consist of three semilunar leaflets, inserted on the annulus in a crown like fashion and meeting at the level of the STJ, forming the commissures (60, 61).

In healthy individuals, the aortic STJ diameter is 10–15% smaller than the annulus diameter and ±25% smaller than the maximal sinus diameter (62, 63). Furthermore, the pulmonary valve diameter is about 2 mm greater than that of the aortic valve and both are closely related in height (64). However, in patients undergoing the Ross operation, the relation between pulmonary artery and aorta may be distorted.

### Microstructure of the Wall and Valve

Both arteries possess a tunica media rich with elastin, endowing the wall with resilience and elastic recoil, and a tunica adventitia consisting primarily of thick collagen fibers, providing strength (58). Compared to the pulmonary artery, the aorta has a thicker wall with a greater content of structural proteins. Furthermore, its tunica media has a greater number of elastic laminae which are more organized with a denser weave (61, 65–67). As most functional elastic fibers are assembled before adulthood, they are susceptible to mechanical fatigue and proteolytic degradation. Collagen fibers, on the other hand, have a short half-life and are subject to constant turn-over in response to changes in wall stress (27). Therefore, *via* the action of mainly fibroblasts, the adventitia plays a crucial role in maintaining mechanical homeostasis. The tunica media consists of concentric elastic laminae interspaced with reticular collagen and smooth muscle cells (SMCs), the latter making up ~35% of the wall by dry weight. Finally, a modest amount of glycosaminoglycans contributes to compressive stiffness and likely mechanosensing. Because the contractile filaments of the SMCs are connected to elastic fibers by focal adhesions, forming so-called elastin-contractile units, the SMCs are the main mechanosensing cells of the tunica media (68). While SMCs are typically considered to have either a contractile or synthetic phenotype, with the latter representing a matrix-remodeling function, it seems that these are two ends of a spectrum (69, 70). Furthermore, the SMC population in the aortic and pulmonary media is inhomogenous with cells from various lineages possessing different matrix-producing abilities in response to changes in wall stress or hypoxia (71).

The aortic and pulmonary leaflets, or cusps, are richly innervated and capable of actively responding to changes in mechanical load (19, 72, 73). The cusps are delineated by endothelium on both the arterial and ventricular side. Their core consists of three layers: the collagen-rich fibrosa on the arterial side, the central spongiosa consisting mainly of glycosaminoglycans, and the ventricularis on the ventricular side, rich in elastin sheets (60). Valvular endothelial cells play an important role in the valve's response to changes in flow-induced shear stress by modulating inflammation, calcification, and ECM remodeling (72, 74). They are different from vascular endothelial cells due to their high proliferation rate, unique gene expression profile and orientation perpendicular to blood flow (72). Valve interstitial cells (VICs), present in all three layers of the leaflet, are the primary matrix remodeling cells that maintain structure and function. The mechanical environment of the VICs is sensed by, amongst others, mechanosensitive ion channels (21). When compared to pulmonary VICs, aortic VICs are stiffer and display a greater ability to contract the ECM (75). Additional functional differences include the greater potential for a pro-inflammatory and pro-osteogenic response in aortic VICs, indicating why valve calcification is common in the aortic valve yet rare in the pulmonary position (76).

### Aortic and Pulmonary Root Biomechanics

The pulmonary autograft undergoes a radical change in environment after the Ross procedure due to differences in hemodynamic conditions. The blood pressure in the aorta is around 120/80 mmHg at rest whereas that in the pulmonary artery is around 25/10 mmHg (77). In the healthy pulmonary and aortic root, blood flow is laminar with sinus vortices behind the leaflets, acting as low-pressure zones to facilitate smooth opening and closing (78, 79). The blood flow acceleration and peak velocity in the aorta are approximately double that of the pulmonary root (80). Furthermore, powerful left ventricular contractions subject the aortic root to cyclic elongation and torsional deformation (81). Sufficient aortic distensibility is required to reduce cardiac workload and provide diastolic coronary flow (82). Cyclic expansion of aortic root volume is nearly twice that of the pulmonary root (37.7 vs. 20.9 %), with the greatest distension occurring at the STJ and commissures (82).

**Figure 5:**
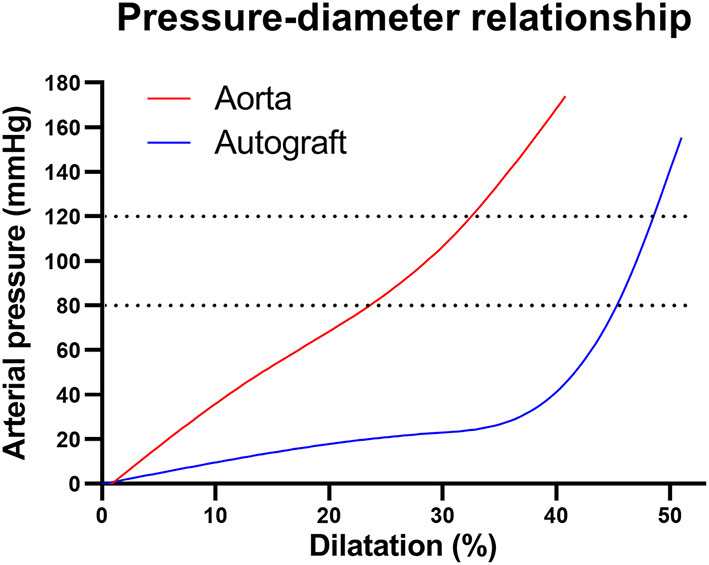
Pressure-diameter behaviors for the healthy aortic and pulmonary root illustrating non-linear mechanical behavior. In the aortic pressure range of 80–120 mmHg (dotted lines), the pulmonary artery behaves very stiff, evident by the steep incline. Figure recreated using data available in the article by Nagy et al. (40).

Both arterial walls display non-linear mechanical behavior and are most compliant in their physiological pressure ranges because their ECM components are deposited and interlinked at vessel-specific levels of transmural pressure and stretch. At physiological arterial pulsatility, the mechanical load is carried mainly by elastic fibers. With increasing distension, collagen fibers are recruited and the artery stiffens (66, 70). As the pulmonary sinuses are most compliant within the physiological transmural pressure range of 0-30 mmHg, the greatest diameter changes are seen in this range (Figure 5). Beyond 30 mmHg, proportionally less distension is seen with increasing pressures as the wall stiffens (40, 83). Therefore, once exposed to systemic pressures after the Ross procedure, and before any remodeling takes place, the autograft wall will behave significantly stiffer than the aorta, evident by a steeper incline of the pressure-diameter curve (Figure 5) (40, 66, 84, 85).

The leaflets of the semilunar valves are exposed to flexural and shear stress in systole and tensile and compressive stress in diastole (1). To accommodate this complex cyclical loading, the leaflets are highly anisotropic: they are stiffer in the circumferential than axial direction, related to the circumferential orientation of collagen fibers as opposed to axially oriented elastin bundles (1, 86). Due to the trans-aortic pressure drop of 60–100 mmHg, the autograft leaflets will suddenly experience a far greater tensile and compressive stress on their arterial surface post-operatively. While the microstructure of pulmonary and aortic leaflets is similar, the latter are typically 50–60% thicker and contain more collagen (87, 88). The mechanical properties of both valves also seem to be similar, yet discrepancies in testing protocols between different studies make it challenging to draw definitive conclusions (86, 87, 89, 90). It seems nonetheless that the pulmonary valve is mechanically sound as an aortic valve substitute, given it is implanted symmetrically with perfect coaptation.

### Implications for Surgical Technique

The relation between annular and STJ dimensions determine leaflet coaptation (39, 43, 44). A dilated aortic annulus (≥27 mm) may indicate an underlying connective tissue problem. Furthermore, in cases of size mismatch, implantation of the autograft within a larger aortic annulus may impart additional pre-stretch. To ensure that the leaflets remain constrained within the aortic annulus, the autograft should be implanted deep within the annulus so that it can benefit from support of the fibrous skeleton of heart. In patients with a large annulus, an external annuloplasty using a band of prosthetic material may be indicated to further stabilize the annulus (19).

There are regional biomechanical differences within both arterial walls: the ascending aorta and main pulmonary artery are more compliant than their respective sinuses due to a greater elastin content (61, 66). Furthermore, because aortic expansion during the cardiac cycle is most pronounced at the level of the commissures, the STJ is at risk for dilatation after the freestanding root Ross (82). The autograft must be trimmed distally, leaving at most 2 mm of pulmonary wall above the commissures. In patients with pre-operative aortic dilatation, the STJ can be stabilized using a prosthetic interposition graft (91). Externally supporting the STJ with a resorbable band of polydioxanone (PDS) has shown to reduce the incidence of neo-aortic regurgitation in children, further confirming the importance of the STJ.

There appear to be no histological or biomechanical differences between the 3 pulmonary sinuses (66). However, during autograft harvesting, the left-facing, septal autograft sinus is stripped of most of its adventitial tissue where it is adherent to the aorta. In the freestanding Ross, this sinus is therefore usually placed in the left coronary sinus so that it benefits from support of the surrounding heart structures (91).

## Conditions Affecting Autograft Properties

The underlying pathology forming the clinical indication for the Ross procedure may affect the pulmonary autograft characteristics. In the general population, degenerative aortic valve stenosis in the elderly is the primary indication for valve replacement (92). BAV disease, characterized by abnormal fusion of the aortic leaflets so that only 2 functional leaflets exist, is of special interest to the Ross procedure. While BAV occurs in 0.5–1.2% of the population, it is present in up to 74% of patients undergoing the Ross procedure as it typically manifests at a younger age than degenerative aortic stenosis (32, 38, 93).

Histological features in the aortic wall of BAV include SMC apoptosis and degeneration of ECM (94). While there is evidence for a genetic basis, increased leaflet stress and abnormal flow patterns influence both the development of BAV and its clinical manifestation (95). For the aorta and pulmonary root, both the leaflets and the cells populating the sinus walls are derived from neural crest and second heart field lineages (96). This provides a developmental link between pathologies affecting the leaflets and sinus walls like the aortic dilatation in up to 50% of patients with BAV disease (97). Neural crest cells, implicated in BAV and congenital aortic stenosis, are less commonly seen in the pulmonary than in the aortic root in murine embryological studies (96). This may explain why anatomic pulmonary valve anomalies, precluding use as an autograft, are rare (incidence of 0.1%) and usually associated with other congenital heart defects (98–100).

While de Sa et al. observed degenerative histological features in the main pulmonary artery of BAV patients (101), the association between aortic and pulmonary degenerative features was not confirmed by other groups (94, 102). Furthermore, when assessed by planar biaxial mechanical testing, there is no difference in mechanical properties of the pulmonary artery according to aortic valve phenotype (67). On the other hand, when assessed *in vivo* by echocardiography, elastic properties of the pulmonary artery are impaired with a correlation between aortic and pulmonary stiffness and diameter in BAV patients (103, 104).

Importantly, the presence of BAV in itself is not a risk factor for autograft failure or dilatation after the freestanding Ross. There are, however, risk factors for dilatation that are associated with BAV and may indicate an underlying connective tissue anomaly, such as aortic insufficiency, a large aortic annulus, or pre-operative aortic dilatation (13, 47, 48). This is in keeping with the observation that the aortic and main pulmonary artery walls of patients with predominantly AR are more compliant than those of patients with predominantly aortic stenosis or mixed stenosis/regurgitation (67). Furthermore, all described cases of autograft dissection occurred in patients with BAV disease. These data highlight the importance of understanding autograft mechanobiology to guide patient selection and predict the risk of dilatation.

Marfan syndrome (MFS) is the most common genetic form of thoracic aortic aneurysm disease, caused by pathogenic variants of the microfibrillar protein fibrillin-1, an ECM component acting as scaffold for elastin, as well as contributing to TGF-β signaling (105). The aorta of patients with MFS is susceptible to dissection or rupture because of its increased stiffness and impaired remodeling ability. MFS and other genetically driven aortopathies are a contra-indication for the Ross procedure because the underlying histologic anomalies, medial degeneration with elastic fiber fragmentation and loss of SMCs may also affect the pulmonary wall (106). Indeed, arterial elastic properties are impaired in MFS, and patients have a larger pulmonary artery diameter with an aneurysm in up to 15.3% (107). If the pulmonary artery of these patients potentially cannot withstand pulmonary pressures, it does not appear fit for use as an aortic autograft.

## Clinical Evidence for Autograft Remodeling

In 2006, Sir Magdi Yacoub wrote: “An evolutionary point in the Ross operation is the inherent capacity of the autograft to adapt to the new environment by altering its structure and physical properties” (108). The potential longevity of the pulmonary autograft, several decades in many patients, strongly supports this notion (17, 18). If the autograft leaflets would not remodel and function simply as passive structures, it is highly unlikely that they would be able to withstand the systemic circulation for several decades.

For the freestanding Ross, it seems that repetitive supraphysiological distension of the pulmonary autograft may lead to progressive wall damage, while also eliciting a mechanobiological response. Clinical explants of autograft walls acquired within the first 3 months after the Ross procedure show SMC loss and fragmented elastic fibers yet also an increase in myofibroblasts (24). Late explants acquired at reoperation for dilatation show fragmentation of elastic fibers and deposition of mucoid material as well as hyperplastic intimal remodeling, marked adventitial fibrosis and an increase in synthetic SMCs and myofibroblasts (24, 25, 109–112). Yacoub et al. investigated dilated autograft wall samples with a mean implantation period of 14.1 ± 4.1 years and contrary to previous studies, they observed a seemingly well-remodeled and revascularized arterial wall with preserved architecture and a mixture of increased organization of elastic lamellae and degenerative features (20). Table 1 provides an overview of the main histological reports evaluating autograft samples acquired from patients and their key findings.

**Table 1:** Overview of the main histological reports evaluating autograft samples acquired from patients and their key findings (20, 24, 25, 109–111, 113).

**References**	**Sample origin**	**Timing**	**Wall**	**Valve**
Goffin et al. (113)	Autopsy after death from ventricular arrhythmia (*n* = 1).	1.5 years	Normal elastin and SMC architecture. Disappearance of dendritic cells.	Thickening of ventricular aspect of leaflets with large numbers of fibroblasts. Disappearance of dendritic cells.
Takkenberg et al. (111)	Reintervention (aortic homograft) for dilatation with AR (*n* = 1).	7 years.	Focal interruption of elastin fibers, intimal hyperplasia, fibrosis.	-
Ishizaka et al. (110)	Reintervention for dilatation with AR (*n* = 4).	1, 3, 4, and 8 years.	Elastin fragmentation, mucopolysaccharide deposition.	-
Rabkin-Aikawa et al. (24)	Reintervention for dilatation with AR (*n* = 4), transplantation (*n* = 3), autopsy (*n* = 2).	3 early (2–10 weeks) and 6 late (2.5–6 years).	Early: elastin fragmentation, granulation tissue. Late: fibrosis, loss of normal SMCs, elastin and collagen without inflammation or calcification.	Trilayered architecture preserved yet leaflets thicker due to pannus with intimal hyperplasia and myofibroblasts on ventricular side of cusp. Reduction of myofibroblast and MMP-13 counts in early vs. late explants.
Schoof et al. (25)	Reintervention for dilatation with AR (*n* = 26), reintervention for AR after subcoronary Ross (*n* = 2), autopsy after freestanding Ross (*n* = 2).	Mean 6.1 ± 3.1 years, range 0.1–11.7 years.	Elastin fragmentation, mucopolysaccharide deposition, adventitial fibrosis, myofibroblast presence and SMC hypertrophy, intimal hyperplasia.	Trilayered architecture preserved yet leaflets thicker due to apposition of extra tissue layer on ventricular side of cusp with intimal hyperplasia, myofibroblasts, collagen and elastin. Similar features in non-failed explant acquired at autopsy. Failed subcoronary implants: grossly disturbed architecture.
Mookhoek et al. (109)	Reintervention for dilatation with AR (*n* = 10), for isolated dilatation (*n* = 1).	Median 11, range 7.3–15.4 years.	-	Trilayered architecture preserved yet leaflets thicker due to apposition of fibrous tissue on ventricular side. Ventricularis contains myofibroblasts and cells positive for MMP1, IL-6 and TGF-β. Increase in collagen fiber density. Evidence of ongoing remodeling at 10 years.
Yacoub et al. (20)	Reintervention for dilatation with AR (*n* = 7), for AR after subcoronary Ross (*n* = 1), autopsy after subcoronary Ross (*n* = 1), autopsy after freestanding Ross (*n* = 1).	Freestanding: mean 14 ± 4 years. Subcoronary: 42 and 44 years.	Mixture of adaptation (increased number of continuous elastic fibers), and disarray (elastin fragmentation and scarce collagen in between). Notable presence of vasa vasorum in outer tunica media and adventitia.	Trilayered architecture preserved yet leaflets thicker due to apposition of tissue on ventricular side, containing elastin, collagen, glycosaminoglycans Thickness of fibrosa layer increased to that of aortic valve. Subcoronary: architecture distorted, calcifications.

*AR, aortic regurgitation; SMC, smooth muscle cell*.

Based on *in vivo* imaging at 1–5 years post-operatively in adults and children who underwent the Ross procedure as a root technique, the pulmonary autograft sinuses appeared significantly stiffer than the native aorta of healthy controls (85, 114, 115). Stiffening of the pulmonary autograft wall upon dilatation is easily explained by the non-linear mechanical behavior of the intramural constituents, and is likely an inevitable early consequence of the Ross procedure. On the other hand, mechanical testing of dilated, “failed” autograft walls shows that they are not only less stiff than healthy aorta, they are also less stiff than normal pulmonary root at aortic and pulmonary pressure ranges, respectively (116, 117). An artery's mechanical behavior *in vivo* is determined by its inherent material stiffness but also by its structural stiffness, related to its anatomical configuration and pre-stretch. As it is uncertain if the described mechanical tests of each unique arterial sample were performed at representative levels of *in vivo* pre-stretch, it is challenging to correlate the mechanical data of these studies with expected *in vivo* behavior. It is unknown if long-term ECM remodeling can restore autograft stiffness to normal values in well-functioning autografts. Furthermore, the long-term implications of wall stiffness on valve and ventricular function remain uncertain.

Several investigators had the opportunity to investigate autograft leaflets explanted at reoperation, transplantation or at autopsy. While leaflets usually retained their typical trilayered architecture, increased leaflet thickness mainly due to the apposition of an extra layer of tissue/pannus on the ventricular side was consistently observed in multiple reports of both failed and well-functioning valves (20, 24, 25, 109, 113). This layer was characterized by intimal hyperplasia, dense collagen and the presence of myofibroblasts and matrix-remodeling enzymes. Even in “failed” autografts, overall leaflet architecture and microstructure were rather well-preserved while the autograft walls exhibited pronounced degeneration. The excellent freedom from reoperation for the subcoronary and inclusion techniques, with some patients surviving 44 years after surgery, support the notion that the pulmonary leaflets may adapt better than the wall (17, 18, 20).

## Experimental Evidence for Autograft Remodeling

One might argue that animal models are not clinically relevant as they often use young, healthy animals and short implantation times compared to the development of clinically relevant autograft dilatation. Nevertheless, they provide the opportunity to study the early adaptation mechanisms in seemingly well remodeled autografts. Table 2 shows an overview of all animal models relevant to the Ross procedure and their main findings. In an ovine model of a main pulmonary artery interposition graft in the descending aorta, pulmonary architecture was well-preserved in some areas while vascular atrophy was observed in others. A proportion of explanted tissue samples exhibited aorta-like mechanical behavior during biaxial tensile testing, indicating the pulmonary artery's ability to remodel (84, 118, 119). In a similar animal model using resorbable external support, Nappi et al. suggest that a “neovessel” developed with an increase in elastic wall components (120). This finding is in stark contrast to findings by Schoof et al., who noted preservation of the typical pulmonary arterial microstructure in pigs (26). While new elastin can be produced during adulthood, it is uncertain to what extent these new fibers can contribute to mechanical adaptation, and to what extent the data by Nappi et al. can be extrapolated to humans (27).

**Table 2:** Overview of animal models relevant to the Ross procedure with main findings (22, 26, 84, 118–124).

**Model**	**References**	**Animal**	**Objective**	**Follow-up**	**Main findings**
Pulmonary (valve) interposition graft in the descending aorta	Lower et al. (124)	Dog	Feasibility study.	Up to 12 months	The pulmonary valve and artery can withstand the systemic circulation.
	Nappi et al. (22, 120)	Sheep	Dilatation, remodeling, resorbable and composite external support.	6 months	Resorbable support prevents excessive dilatation, enables diameter increase in proportion to somatic growth. Wall erosion underneath stiff materials.
	Vanderveken et al. (118, 119)	Sheep	Dilatation, remodeling, mechanical properties, porous mesh support.	6 months	Remodeling in line with earlier studies. Support halts dilatation yet with risk of vascular atrophy. Mechanical adaptation in some samples.
Ross procedure	Pillsbury et al. (125)	Dog	Feasibility study.	12–14 months	The subcoronary Ross procedure is technically feasible.
	Schoof et al. (26, 121)	Pig	Dilatation in growing animals, tissue remodeling.	10 months	Increase in size along with somatic growth. Wall: revascularized, typical architecture preserved, SMC's enlarged, collagen increase. Valve: enlarges more than can be explained by merely somatic growth.
	Tudorache et al. (122)	Sheep	Dilatation, valve function, cellular characterization.	20 months	Valve: native cell distribution, neovascularization in leaflet base, trilayered architecture preserved.

*SMC, smooth muscle cell. Adapted with permission from Van Hoof et al. (123)*.

**Figure 6:**
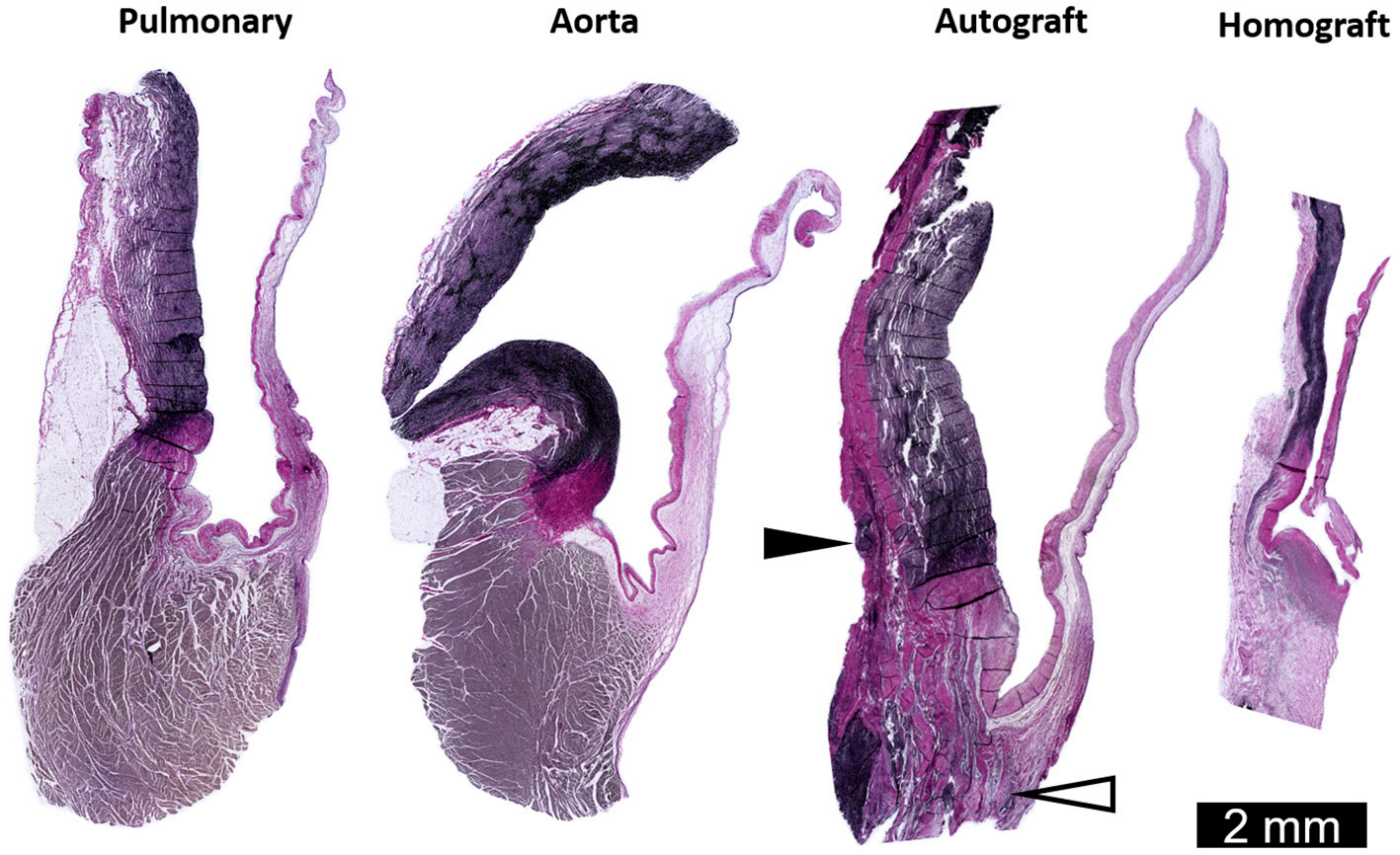
Representative longitudinal sections through the sinus and leaflets of the pulmonary artery and aorta of a sheep weighing 60 kg. Also shown are the pulmonary autograft and homograft at 6 months post-operatively in a sheep who underwent the Ross procedure (weighing 43 kg at operation). Neo-vascularization in the base of the pulmonary autograft leaflet (white arrowhead) and added collagen on the adventitial side of the sinus wall (black arrowhead). The arterial wall and leaflet of the pulmonary homograft are thin and acellular. Elastica Von Gieson staining. Adapted with permission from Van Hoof et al. (123).

In a porcine model of the freestanding Ross procedure, Schoof et al. reported a revascularized wall lacking degenerative features after 10 months. Furthermore, enlarged and rearranged SMCs were seen alongside adventitial fibrosis (26, 121). In the longest experimental evaluation to date, Tudorache et al. observed preservation of typical tri-layered leaflet histology with normal cellular distribution at 22 ± 2.7 months in lambs after the freestanding Ross procedure. Unfortunately, they did not investigate the dilated autograft wall or ECM remodeling of the valve (122). Our group recently developed an ovine model of the Ross procedure performed as a freestanding root replacement (123). Preliminary histological evaluation is consistent with previous clinical and experimental explant studies (Figure 6). Future work will include a comprehensive evaluation of autograft mechanobiology with analysis of hemodynamic parameters, imaging, histology, gene expression response and mechanical testing of explanted tissues.

## Discussion

### Understanding Pulmonary Autograft Adaptation

#### ECM Damage and Remodeling

**Figure 7:**
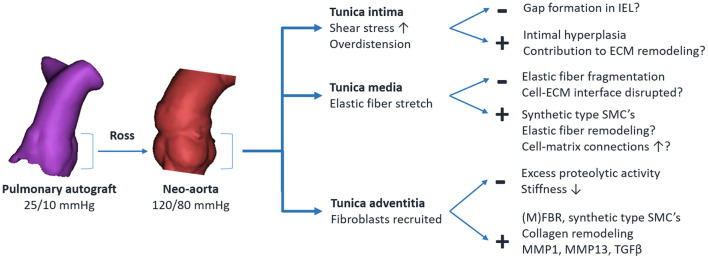
Overview of established and potentially involved mechanisms of pulmonary autograft wall remodeling in the Ross procedure. The + indicates an adaptive response, — indicates maladaptive remodeling. IEL, internal elastic lamina; SMC, vascular smooth muscle cell; ECM, extracellular matrix; MMP, matrix metalloproteinase; TGFβ, transforming growth factor β; (M)FBR, fibroblast/myofibroblast.

Insight into the underlying mechanisms can be gained by correlating established principles of arterial mechanobiology with changes observed in the pulmonary autograft. In physiological conditions, SMCs and adventitial fibroblasts are stress-shielded mainly by elastic fibers. Upon completion of the freestanding Ross procedure, the autograft wall dilates, resulting in a 5- to 10-fold greater circumferential wall stress (126). As the elastic fibers become stretched and the mechanical load is transmitted to previously underrecruited collagen fibers, the artery behaves stiffer (28). This disruption of baseline stress values prompts a response to restore mechanical homeostasis. *Via* cell-ECM and cell-cell connections, cells sense the stress acting on the ECM. Changes in the mechanical environment are translated into intracellular signals *via* specific mechanotransduction pathways, inducing the production of ECM proteins, adhesion molecules and matrix metalloproteinases (MMPs) (70). Figure 7 provides an overview of known and potentially involved mechanisms of ECM remodeling.

Fibroblasts located mainly in the adventitia are among the first to be activated by overdistension (127). These fibroblasts proliferate, and *via* the release of growth factors, induce the differentiation of SMCs from the contractile to the synthetic phenotype. Fibroblasts differentiate into myofibroblasts by the influence of transforming growth factor β (TGF-β) and mechanical stresses (69, 128). Collagen is deposited at the newly imposed mechanical load while existing fibers are removed by MMPs or modulated, for example by changing collagen cross-linking patterns (27). Increased adventitial collagen is observed in all autograft samples acquired at reoperation and in all animal studies, suggesting a common mode of remodeling in both failed and well-adapted autografts. This form of ECM remodeling is a crucial part of arterial homeostasis in arterial aging, pulmonary arterial hypertension and aneurysm development (27, 129, 130). Elevated myofibroblast, MMP1, MMP13 and TGF-β levels are seen in clinical autograft explants at over 10 years post-operatively (24, 25, 30, 109). This persistent ECM remodeling likely indicates the inability to restore the homeostatic state.

As regeneration of elastic fibers is limited, certainly in comparison with collagen, they are susceptible to mechanical fatigue by repetitive overdistension. Currently, the threshold pressure or diameter which causes acute elastic damage is unknown. In an *ex vivo* inflation set-up, the main pulmonary artery became susceptible to damage upon acute exposure to a transmural pressure beyond 60 mmHg, with pressures above 100 mmHg likely to induce collagen damage as well (131). Because this study by Wang et al. employed previously frozen porcine pulmonary arteries, it is uncertain if these data can be extrapolated to the Ross procedure in humans. Previous studies of dilated, explanted pulmonary autografts show fragmented elastin fibers and an increase in synthetic-type smooth muscle cells. As the stress in SMCs is typically concentrated at focal adhesions, connecting the cytoskeleton to surrounding elastic fibers, it seems likely that damage to the elastic-contractile units occurs (68). Besides representing a change in mechanical properties, elastic fiber fragments have a signaling function by inducing SMC proliferation and a phenotype switch, and will undergo proteolytic degradation by MMPs and cathepsins (28, 132).

Immediately after the Ross procedure, autograft leaflets are exposed to elevated shear, compressive and tensile stresses. Furthermore, aortic root dilatation may contribute to cusp stretching. It is likely that collagen production plays an important role in valve remodeling in response to increased stress, as it does in the wall, with an important role for VICs in the cusps (133). Previous histological studies suggest successful leaflet adaptation to the systemic circulation, with increased cusp mass mainly due to the apposition of an extra layer of fibrous tissue on the ventricular cusp side (20, 24, 25, 109). While this suggests improved mechanical properties, there are no data to support this. The absence of the degenerative features which are seen in dilated autograft walls could indicate a greater ability of the leaflet to adapt to the systemic circulation. By preventing excessive deformation and providing reciprocal support, good leaflet coaptation in the early phase after the Ross procedure likely protects the cusps from acute ECM damage, thereby permitting adaptation. Endothelial to mesenchymal transition may also play a role in maintaining leaflet integrity (1).

#### Blood Flow, Shear Stress, and the Endothelium

Endothelial hyperplasia is consistently observed in wall samples explanted late after the Ross procedure (20, 25). This feature, commonly seen in aortic aneurysms, suggests remodeling in response to elevated shear or intramural stress (134). Furthermore, endothelial cells of explanted autograft valves express Ephrin B2. While this may indicate a stable systemic VEC phenotype, it cannot be excluded that these cells migrated from the native aorta or endocardium (24).

Among all aortic valve substitutes, blood flow patterns are closest to normal after the Ross operation (7, 41). In BAV disease, abnormal flow patterns with elevated wall shear stress associate with aneurysm progression and focal elastin degeneration (135). The changes in flow and shear stress that occur in the pulmonary autograft, upon transposition from pulmonary to aortic position, have not been quantified. Therefore, the role of abnormal flow patterns, pre-existent or related to neo-aortic regurgitation which develops later, on autograft remodeling remains unknown.

#### Ischemia and Inflammation

As their wall is thicker than 0.5 mm, the external layers of the pulmonary sinuses are supplied by vasa vasorum (136). Disruption thereof during autograft harvesting may lead to damage or impair remodeling. Both in a porcine model of the Ross procedure and in clinical explant studies, a well-revascularized arterial wall with normal to slightly increased presence of vasa vasorum in the tunica media and adventitia was seen (20, 25, 26). The magnitude of this contribution and the role of neovascularization in mechanobiological adaptation after the Ross procedure are unknown. The autograft leaflets are likely less affected by ischemia as they are supplied by diffusion from both the aortic and ventricular side.

Goffin et al. observed a disappearance of dendritic cells in an explanted autograft of a 14-year old patient who died of unrelated causes (113). While these accessory immune cells play a role in regulating early ECM remodeling, the significance of this finding is uncertain (127). *In vitro*, repetitive overstretching of valvular endothelial cells leads to an upregulation of inflammatory pathways (74). However, there is no evidence for relevant inflammatory activity or calcification in samples explanted several years after the Ross procedure.

#### Molecular Mechanisms Warranting Further Research

A recent study by Chiarini et al. suggests a unique maladaptive process in the autograft which differs from that of aortic aneurysms (30). They compared the proteomic signature of the tunica media of dilated autografts, acquired 8–16 years post-operatively, against that for normal PA, aorta and aortic aneurysm samples. An upregulation of paxillin, a key component of focal adhesions, was observed. As the integrin-containing focal adhesions mechanically link the ECM and actin cytoskeleton, their finding may represent abnormal cytoskeleton remodeling and impaired mechanotransduction (70). Vimentin, a component of the SMC cytoskeleton, was also upregulated, confirming the activation of pathways involving synthetic SMCs. A downregulation of MAGP1 was seen, suggesting impaired elastic fiber buildup. They also found evidence of a disturbed JAG1-Notch1 signaling, potentially impeding tissue remodeling by limiting cell-cell interactions. Unfortunately, it is unknown if the observed proteomic changes represent a cause or consequence of maladaptation and repetitive overdistension.

In the general population, pulmonary artery aneurysm is a rare entity, reported only 8 times in 109,571 autopsies and mainly associated with pulmonary arterial hypertension in the setting of congenital heart disease (130, 137). There are yet several molecular pathways implied in thoracic aortic aneurysms which are also involved in the development of pulmonary arterial hypertension and pulmonary artery aneurysm. Analogous with BAV disease, a combination of hemodynamics and underlying molecular pathways likely impair mechanoregulation in both the aorta and pulmonary artery (130). With regards to collagen integrity, deficiencies in the following proteins are relevant: biglycan (an ECM component regulating collagen formation) and collagen 3 α1 chain (implied in Ehlers-Danlos Syndrome type IV). For elastic fibers: fibrillin-1 (elastic fiber core glycoprotein with signaling function, implicated in MFS) and fibulin 4 (regulation of elastic fiber assembly). Reduced expression of lysyl oxidase, a collagen and elastin cross-linking enzyme, also leads to mural degeneration. For the actin cytoskeleton, proteins like smooth muscle α-actin and filamin A are relevant. With regards to mechanoregulation, TGF-β receptors (implicated in Loeys-Dietz syndrome) and Notch1 (impaired signaling seen in dilated autografts) are implicated (130). These interesting associations provide insight into possible underlying mechanisms of pulmonary autograft maladaptation. Finally, the role of mechanosensitive ion channels should be explored (21).

### Strategies to Prevent Pulmonary Autograft Dilatation

#### Antihypertensive Treatment

Undoubtedly, blood pressure is one of the main determinants of remodeling. As early remodeling is crucial to avoid initiating a vicious cycle of pathological remodeling, strict blood pressure control (systolic pressure <110 mmHg) is advocated by many experts starting immediately post-operatively and continued for 6–12 months (19). While there is currently no direct evidence for an effect on dilatation, reoperation rate or histological outcome, aggressive blood pressure control appears justified as there are many indirect arguments that antihypertensive medication can improve autograft remodeling. In ascending aortic aneurysms, hypertension is associated with aneurysm growth rate as well as dissection (138). While the decrease in wall stress is non-linearly related to blood pressure lowering, antihypertensive treatment can achieve a marked reduction in wall stress, even in patients with mildly elevated blood pressure. Therefore, even in patients with normal or slightly elevated blood pressure, antihypertensive treatment can achieve a marked reduction in wall stress. In the Ross procedure, this could make the difference needed to allow autograft adaptation.

The underlying idea is to gradually expose the autograft to aortic pressures. There is anecdotal evidence that preoperative pulmonary arterial hypertension pre-conditions the autograft by promoting ECM organization (65). In animal models of pulmonary hypertension, thickness of the tunica media is increased (29). This concept is further supported by numerical simulations of saphenous vein remodeling when used as arterial bypass graft, whereby gradual loading improves remodeling (139).

In both MFS and after the Ross procedure, a repetitive cycle of overdistension occurs in an arterial wall at risk for pathological remodeling (140). Aortic stiffness is inherently increased in MFS. Similarly, the pulmonary autograft wall behaves stiffer than the aorta at systemic pressures, at least before remodeling (84). By lowering blood pressure and the force and velocity of ventricular contraction, beta-blockers reduce aortic wall stress, thereby lowering the risk of aortic dissection in patients with MFS and potentially enabling the wall to heal. Angiotensin-converting enzyme inhibitors and angiotensin II receptor blockers reduce blood pressure and aortic stiffness (105). In murine models of MFS, prenatal initiation of angiotensin II receptor blockers has the potential to prevent pathological remodeling and aortic dilatation. As this effect is related to hemodynamic and vasomotor changes as well as the interference with TGF-β signaling, it may be of interest to the Ross procedure (141).

#### Mechanobiology of External Autograft Support

The technical considerations of currently used strategies to prevent autograft dilatation were recently reviewed by Chauvette et al. (23). While it is uncertain at this point what the best strategy is, external support should be biocompatible in terms of geometry, compliance and tissue reaction. The primary goal of external support is to stabilize root dimensions; this will inevitably reduce distensibility, and therefore circumferential wall stress and strain. Repetitive strain is crucial to arterial homeostasis, just as mechanical loading is essential for the maintenance of bone density and skeletal muscle mass (142). In a landmark study, Courtman et al. banded the infrarenal abdominal aorta in rabbits, thereby reducing local strain. At 6 weeks, they observed apoptotic loss of 30% of SMCs and a 45% reduction of medial area. No endothelial cell loss was seen, nor was neo-intimalization impaired in animals undergoing banding and endothelial balloon denudation (143). In an experiment involving iliac artery wrapping in baboons, a tighter wrap induced more ECM loss and SMC atrophy (144). These banding studies indicate that excessive stress-shielding, so that cells experience low tensile stress, is undesirable. This is analogous to applying a tight cast to a fractured leg without allowing movement of the limb, resulting in muscle and bone atrophy (142). Furthermore, damage induced by compression between the wrap and pulsatile blood flow cannot be excluded. Numerical simulations of prosthetic external support of the descending aorta indicate the potential of external support to ameliorate the arterial homeostatic response to elevated pressure, and also point to stiffness of the support material as a crucial determinant of remodeling (145). It seems that the autograft needs to feel just the right amount of stress in order to heal after being devascularized during the operation, and subsequently remodel.

Any type of prosthetic external support induces a tissue reaction, influenced by the material, presence of a coating and porosity. Early cellular inflammation soon shifts to a foreign body giant cell reaction, characterized by macrophages and giant cells attempting to encapsulate the implanted material (146). For external support, porosity is one of the most important parameters. A porous material will permit the ingrowth of a fibrotic neo-adventitia and blood vessels, thereby enabling the transport of oxygen and nutrients through the material (147, 148). Furthermore, the graft will become anchored to the arterial wall, forming a composite. A non-biodegradable low-porosity material, on the other hand, will prevent tissue ingrowth and elicit the formation of a thick sheath of fibrosis, potentially further increasing arterial stiffness (148, 149). As the inflammatory reaction induced by the graft is situated in the peri-adventitia, its contribution to vascular remodeling is unknown.

##### Autologous Inclusion Technique

In the autologous inclusion technique (Figure 1.3), the autograft is sutured into the aortic annulus and then included within the native aortic wall, which is reduced or enlarged to achieve the desired dimension (18). From an anatomical and biomechanical point of view, this technique optimally preserves functional aortic root integrity and compliance. The native aorta naturally augments the autograft's structural properties to withstand systemic pressures, though without a detailed analysis of changes in wall stress from homeostatic values. While this technique is technically challenging, an exceptionally low incidence of significant root dilatation and reoperation can be achieved (18). To our knowledge, there are no histological reports of autografts explanted after the inclusion technique.

##### Dacron Vascular Prosthesis

**Figure 8:**
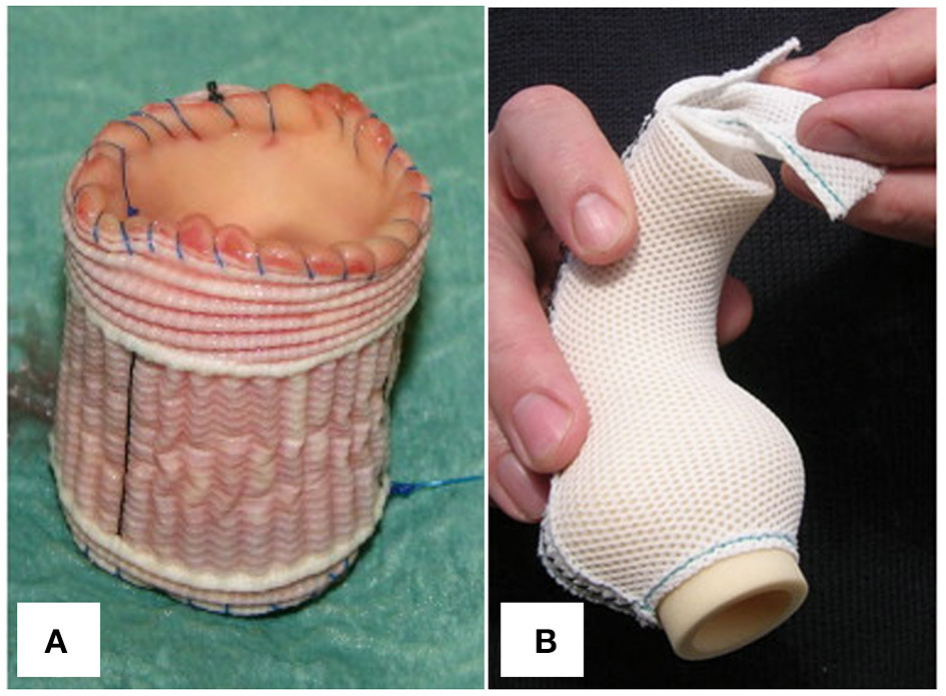
**(A)** Pulmonary autograft wrapped with a cylinder of microporous Dacron graft. **(B)** Personalized external aortic root support implant fashioned from porous, soft mesh. Figure adapted with permission from Carrel et al. (34) and Treasure et al. (150).

The autograft can be included within a segment of Dacron vascular tube or Valsalva graft before implantation into the aortic annulus (Figure 8A), stabilizing neo-aortic dimensions up to 5 years post-operatively (34–36). While this may seem a straightforward and reproducible technique, it risks distorting the autograft and preventing it from settling into its natural post-operative shape. As a microporous and sealed graft allows limited tissue ingrowth, seroma formation, erosion and graft migration have been a great concern for conventional aortic wrapping of aneurysms using the same material (151, 152). Because the rigid, woven material impairs pulsatility, there are also concerns about elevated leaflet stress and abnormal flow patterns, as evidenced by 4D MRI studies after valve-sparing root replacement (79). Finally, vascular atrophy seems inevitable with this stiff material due to excessive stress-shielding (148). To date, these complications have not been reported for the supported Ross procedure.

##### Personalized External Aortic Root Support

In Personalized External Aortic Root Support (PEARS), a soft, custom-made mesh is used to stabilize a moderately dilated aortic root with at most mild AR, primarily in patients with MFS (153). The porous support (Figure 8B) becomes well-incorporated into a neo-adventitia and stabilizes root dimensions (148, 150, 154–156). Subsequently, the risk of dissection is mitigated by avoiding repetitive overdistension. Pepper et al. had the opportunity to histologically examine the aorta of a patient with MFS who underwent PEARS and later died of unrelated causes. The supported region of his aorta showed a normal tunica media whereas the unsupported aortic arch showed features of Marfan syndrome, suggesting that the external support allowed the aorta to heal (156).

This flexible, porous material that conforms to aortic anatomy holds great promise for the Ross procedure and has currently been applied in nearly 50 patients (157). In conventional aortic PEARS, the support is modeled using the patient's pre-operative aortic CT scan. The challenge in the Ross procedure has been to predict external support morphology based on pre-operative imaging, as the pulmonary autograft changes shape and dilates immediately upon exposure to aortic pressures. In an ovine model of a pulmonary artery interposition graft in the descending aorta, the mesh material used in PEARS successfully stabilized dimensions at 2 months after allowing some initial dilatation. All supported samples showed thinning of the media with SMC atrophy (119). While this atrophy may be an inevitable consequence of stress-shielding, it indicates that improvements to the material's mechanical properties are possible. Furthermore, a major difference with conventional aortic PEARS is that the pulmonary autograft is devascularized during the operation, and external support may influence healing and the development of vasa vasorum.

##### Resorbable Materials

If protection during the early phase, when the risk of dilatation is greatest, will promote appropriate pulmonary autograft remodeling, external support might not be required permanently. One could envision a composite external support, partially consisting of resorbable material, gradually resorbing and allowing some initial dilatation while the degree of support decreases. A similar strategy is used to promote *in vivo* neovessel development using biodegradable tissue engineering scaffolds (158).

The clinical use of resorbable support was already described in 1993 by Moritz et al., who used a VICRYL® mesh composed of polyglactin 910. Unfortunately, data on the effect on dilatation is not available (159). Nappi et al. designed a composite support consisting of a resorbable polydioxanone mesh interwoven with fibers of expanded polytetrafluoroethylene, the latter allowing gradual expansion but serving as permanent support. They used this material as support for a pulmonary artery interposition graft in the descending aorta of 10 growing sheep. At 6 months, dilatation was effectively prevented and the material was well-incorporated without marked inflammatory changes (22). Unfortunately, their report lacks reliable measurements of wall thickness, protein fractions, elastin integrity and mechanical testing to confirm remodeling. While composite resorbable support holds great promise to prevent neo-aortic root dilatation, like in the Ross procedure, its clinical implementation is currently not justified.

### Remaining Questions and Future Perspectives

#### Understanding and Promoting Pulmonary Autograft Remodeling

The available evidence indicates that the pulmonary autograft can become a permanent solution for a wide range of patients. Several fundamental questions remain to be answered before this can become reality (Table 3). Besides the universally observed collagen remodeling by myofibroblasts and synthetic type SMCs, it is unknown which mechanoregulation pathways enable some autografts to withstand systemic conditions for several decades. Candidate mechanisms include changes in collagen cross-linking or an increase in cell-ECM connections.

**Table 3:** Remaining fundamental questions and unmet clinical needs regarding pulmonary autograft remodeling after the Ross procedure.

**Understanding pulmonary autograft remodeling**
**Mechanotransduction and -regulation mechanisms in wall and valve**
Established mechanism: collagen deposition
Additional mechanisms which determine (mal)adaptation?
Cell-cell or cell-ECM adhesions
Collagen cross-linking
Role of endothelial cells, shear stress and blood flow?
**Timeline of adaptation? Is it ever complete?**
**What defines (mal)adaptation?**
Aorta and PA have common embryological origin –> Can the autograft truly develop aorta-like microstructure and mechanical properties?
Have they diverged too far apart? Is any observed remodeling merely a coping mechanism, leading to a new equilibrium at best?
Risk of dissection in dilated autograft—criteria for reintervention?
**Are pulmonary leaflets better suited than wall to withstand systemic**
**conditions?**
Different innate remodeling ability? Related to distinct mechanical loading?
**Strategies to improve autograft longevity and adaptation**
**Ideal conditions for autograft?**
Geometry: sinus/cusp orientation, proportions annulus-sinus-STJ, …
How much stress is ideal/acceptable for wall and leaflet?
Blood pressure target?
How much autograft wall dilatation is desirable or can be tolerated?
Threshold for damage—start of pathological cycle of remodeling?
**Optimizing patient selection**
Can we go beyond anatomical and demographic variables?
***In vivo*** **quantification of arterial properties and autograft (mal)adaptation**
Mechanical properties: stiffness, elasticity > Imaging
Biological processes: collagen cross-linking, … > Biomarkers
> Pre-operative: predict dilatation, guide patient selection
> Post-operative: identify maladaptation, risk of reoperation/dissection
**External support**
Can we reduce the reintervention rate without collateral damage?
Risk of erosion, seroma formation, graft migration?
Effect on LV and leaflet stress?
Ideal material properties? Role for resorbable materials?
Outcome of PEARS for the Ross operation?
**Antihypertensive treatment**
Effect on reintervention rate?
Hemodynamic effect vs. direct influence on remodeling pathways?
Other strategies to pharmacologically influence remodeling?

A greater understanding of the relation between patient characteristics and autograft remodeling may identify biomarkers or cardiac imaging features related to autograft maladaptation. This will enable us to define in which patients the autograft's innate remodeling ability will likely suffice to withstand systemic conditions, and in whom additional measures are needed to guarantee adaptation. A pre-operative *in vivo* assessment of the pulmonary autograft using dynamic imaging studies may indicate whether a patient is a good candidate for the Ross procedure. Serial post-operative imaging studies evaluating changes in stiffness may identify patients at risk for excessive dilatation or reoperation (85, 114, 115). As in aortic aneurysms, localized changes in shear stress may signal underlying elastin degradation (135). In patients with congestive heart failure, excessive myocardial collagen cross-linking indicates undue cardiac stiffness and associates with adverse clinical outcome. This collagen cross-linking can be quantified non-invasively on blood samples or based on the urinary proteome (160). Furthermore, diastolic left ventricular function can be predicted based on the urinary proteome in individuals without heart failure (161). Similarly, serial biochemical studies in patients before and after the Ross procedure might identify biomarkers to guide patient selection or confirm the presence of maladaptation in the post-operative setting.

*In vitro* culture of a pulmonary artery in a bioreactor may provide valuable insights into the timeline of early remodeling and the role of shear stress, pre-stretch at implantation and acute hypertension. Large animal models also hold great promise to evaluate whether current strategies to prevent dilatation—external support and antihypertensive treatment—promote remodeling, either by modifying the mechanical environment or by interfering with molecular pathways (123). To assess whether the autograft remains viable—capable of healing and regulating its mechanical properties—active mechanical testing of freshly explanted leaflets and wall would be required (162). A rat model with heterotopic implantation of the pulmonary root of a syngeneic donor animal into the abdominal aorta would allow a serial evaluation of adaptation in the first post-operative months at a lower cost.

By providing data on microstructure, mechanical properties, geometry, hemodynamics and the underlying pathways, experimental models can enable numerical simulation of autograft remodeling (163, 164). Subsequently, the ideal conditions for remodeling, or conversely, the risk of dissection, could be identified in so-called *in silico* trials (165). Until now, available computational studies have mainly confirmed important *in vivo* observations, such as the importance of STJ dimensions on wall and leaflet stress (126, 163, 166, 167). It has proven very challenging to mathematically simulate the complex torsional deformation of the aortic root which results in spiraling blood flow and flow vortices behind leaflets (41).

#### Preventing Autograft Dilatation in the Freestanding Ross Procedure

Notwithstanding its clinical use since 2004, several questions about prosthetic external support of the autograft sinuses should be resolved (36). First, concerns about the deleterious hemodynamic effects on the left ventricle and autograft leaflets should be addressed by echocardiographic and 4D flow MRI studies. Second, once placed in the aortic position, optimal autograft geometry must be clearly defined. Quantitative comparison of pre-operative and post-operative imaging in patients undergoing the Ross procedure might yield a predictive algorithm for ideal autograft geometry and subsequent external support configuration.

The mechanical properties of external support should be determined by how much stress-shielding is needed in each phase of remodeling. To this end, the timeline of changes in mechanical properties should be evaluated in clinical imaging studies and in an animal model. Finally, polymer materials are required which truly augment the autograft. Even the best currently used polymer materials, including resorbable ones, are relatively stiff and inelastic (119). Therefore, the mechanical behavior will be dominated by the external support, potentially leading to excessive stress-shielding and deleterious hemodynamic effects. Polyglycerol sebacate (PGS) is a promising new resorbable elastomer which has been used as tissue-engineered vascular grafts to serve as scaffold for cellularization (168). More futuristic options include a bioengineered matrix of collagen and elastin, containing growth factors, or even seeded with stem cells, immediately optimizing mechanical properties while promoting autograft incorporation into the aortic root (169).

## Conclusions

Long-term clinical success of the Ross procedure relies on a well-functioning, living valve integrated into an aortic root having a normal hemodynamic profile. From this point of view, the subcoronary and autologous inclusion technique may be superior to the freestanding root technique, yet the latter is applicable over a wider spectrum of cardiac anatomy and is more surgically reproducible. All evidence indicates that the autograft valve is suited to withstand the systemic circulation and remodel, given that it is implanted symmetrically with stable annular and STJ dimensions over time. Therefore, one of the key surgical principles of the Ross procedure is to ensure that the proximal autograft is constrained within the native aortic annulus. The autograft sinuses, on the other hand, sit unrestrained after a freestanding Ross procedure and are at risk of dilating and subsequently causing the valve to fail. The currently available evidence indicates that the pulmonary autograft wall is not capable of truly achieving mechanical homeostasis and remodeling into an aortic phenotype. Perhaps the aortic and pulmonary root have diverged too far apart after arising from a common embryological origin.

Protecting the autograft during the early adaptation phase is crucial to avoid initiating a sequence of pathological remodeling. Therefore, strict blood pressure control during the first 6–12 post-operative months is justified to reduce wall and leaflet stress. Adequate patient selection is critical and the surgical technique should be tailored individually, aiming to minimize the amount of wall tissue exposed to aortic pressures. While external autograft support may stabilize root dimensions, its efficacy should be measured by a reduction in reinterventions, without negatively affecting valve or left ventricular function.

Remodeling of the ECM with mainly the production of additional collagen is a common feature in both autograft walls and leaflets. A distinct feature of autograft leaflets is the apposition of an extra layer of tissue on the ventricular side, resulting in increased leaflet thickness. Future studies should pinpoint the remodeling processes in well-remodeled and externally supported autografts. Several molecular pathways are proposed. To this end, animal models or bioreactor studies should include a comprehensive mechanobiological assessment at different time-points consisting of microstructural evaluation, transcriptional and proteomic characterization, mechanical testing of tissue samples and dynamic imaging studies with complete hemodynamic profile.

Numerical simulations of tissue growth and remodeling may aid in distilling the ideal conditions for autograft adaptation. Subsequently, a patient-specific strategy for autograft protection and external support could be determined, and the indications for the Ross procedure might be expanded. Widespread clinical implementation of the PEARS concept for the Ross procedure is greatly anticipated because of the many advantages over microporous vascular grafts. Future innovations to external support may include the use of resorbable materials or bio-engineered scaffolds to augment the autograft's mechanical properties and guide remodeling.

## Author Contributions

This manuscript is the result of 3 years of research into the mechanobiology of the Ross procedure in the context of the PhD program of LV, performed under supervision of FR, PV, NF, and EJ. LV wrote the first draft and edited the figures and tables. Subsequent revisions were written based on multidisciplinary discussions involving LV, PV, EJ, JH, SJ, NF, and FR, including written feedback. All authors approved the final version.

## Funding

KU Leuven Research Project (C2 Project: C24/16/026 24ZKD1128-00-W01) + LV is the holder of a predoctoral grant Strategic basic research (SB 1S70220N) from the Research Foundation Flanders (FWO).

## Conflict of Interest

The authors declare that the research was conducted in the absence of any commercial or financial relationships that could be construed as a potential conflict of interest.

## Publisher's Note

All claims expressed in this article are solely those of the authors and do not necessarily represent those of their affiliated organizations, or those of the publisher, the editors and the reviewers. Any product that may be evaluated in this article, or claim that may be made by its manufacturer, is not guaranteed or endorsed by the publisher.
